# A kinase-independent function for AURORA-A in replisome assembly during DNA replication initiation

**DOI:** 10.1093/nar/gkaa570

**Published:** 2020-07-11

**Authors:** Estrella Guarino Almeida, Xavier Renaudin, Ashok R Venkitaraman

**Affiliations:** The Medical Research Council Cancer Unit, University of Cambridge, Hills Road, Cambridge CB2 0XZ, UK; The Medical Research Council Cancer Unit, University of Cambridge, Hills Road, Cambridge CB2 0XZ, UK; The Medical Research Council Cancer Unit, University of Cambridge, Hills Road, Cambridge CB2 0XZ, UK

## Abstract

The catalytic activity of human AURORA-A kinase (AURKA) regulates mitotic progression, and its frequent overexpression in major forms of epithelial cancer is associated with aneuploidy and carcinogenesis. Here, we report an unexpected, kinase-independent function for AURKA in DNA replication initiation whose inhibition through a class of allosteric inhibitors opens avenues for cancer therapy. We show that genetic depletion of AURKA, or its inhibition by allosteric but not catalytic inhibitors, blocks the G1-S cell cycle transition. A catalytically inactive AURKA mutant suffices to overcome this block. We identify a multiprotein complex between AURKA and the replisome components MCM7, WDHD1 and POLD1 formed during G1, and demonstrate that allosteric but not catalytic inhibitors prevent the chromatin assembly of functional replisomes. Indeed, allosteric but not catalytic AURKA inhibitors sensitize cancer cells to inhibition of the CDC7 kinase subunit of the replication-initiating factor DDK. Thus, our findings define a mechanism essential for replisome assembly during DNA replication initiation that is vulnerable to inhibition as combination therapy in cancer.

## INTRODUCTION

Human cancers originating in many different tissues frequently amplify or overexpress the *AURKA* gene (1–3), but how the 403 amino acid protein kinase encoded by the gene promotes carcinogenesis remains unclear. Consistent with normally elevated expression during the G2 and M phases of the cell cycle, AURKA has been implicated as a key regulator of mitotic chromosome segregation through its functions in chromosome condensation, mitotic spindle assembly and the bipolar attachment of kinetochores to spindle microtubules, as well as entry into and exit from mitosis (4–9). Furthermore, AURKA-mediated phosphorylation of the replication licensing factor geminin on Thr25 during M phase has been reported. This event induces geminin stabilization by preventing its APC/C ubiquitin ligase complex-mediated degradation, ensuring its persistence during the M-G1 transition (10). However, recent evidence suggests that AURKA is also expressed during the G1 and S phases of the cell cycle and in non-cycling cells, where it has been implicated in different non-mitotic processes (11), including protection of DNA forks during replication stress (12), regulation of the expression of the DNA damage-response genes BRCA1, CHK2 or BRCA2 (13,14), the disassembly of primary cilia during cell cycle entry (15,16), or control of mitochondrial dynamics and energy production (17). Moreover, accumulating evidence suggests that AURKA exerts functions that are kinase-independent, as well as through its catalytic activity. For instance, defects in mitotic spindle assembly induced by AURKA depletion are rescued by a kinase-dead catalytic mutant (18), and a similar catalytically-inactive mutant is capable of transactivating transcription driven by the MYC oncogene (19). In addition to kinase activity, which is targeted by small-molecule inhibitors now in clinical use (20,21), discrete AURKA protein conformations and protein–protein interactions that also underlie its biological functions have been identified. AURKA interacts with the mitotic protein TPX2, inducing an allosteric change that activates kinase catalytic activity, which is blocked by small-molecule inhibitors of the AURKA/TPX2 interaction (22–24). Likewise, a conformation of AURKA that interacts with the N-MYC protein can be blocked by allosteric (but not catalytic) small-molecule inhibitors, suppressing N-MYC activation in neuronal cancer cells (25).

Although AURKA has been associated with geminin phosphorylation during mitosis to prevent its degradation (10), no relationship between AURKA and the replication machinery in interphase has been described to date. Here, we have deployed both catalytic and allosteric inhibitors of AURKA to reveal a previously unrecognized non-catalytic function of the protein in DNA replication. We demonstrate that AURKA is necessary for the assembly of functional replisomes during the G1-S phase transition, and that allosteric but not catalytic inhibitors prevent the chromatin loading of replication factors required for the efficient initiation of DNA replication. We also show that allosteric but not catalytic AURKA inhibitors sensitize cancer cells to inhibition of the CDC7 kinase subunit of the replication-initiating factor DDK. Thus, our findings provide fresh insights into the mechanisms that control human DNA replication, and suggest an approach for combination therapy in cancer.

## MATERIALS AND METHODS

### Cell lines and reagents

Parental FRT/TO HeLa (kind gift from Stephen Taylor, University of Manchester), A569 (adenocarcinomatous human alveolar basal epithelial cells) and EUFA423 (fibroblast derived from Fanconi anemia subtype D1 patients) were grown in DMEM (Gibco) medium, SW48 (colorectal adenocarcinoma cell line) was cultured in RPMI (Gibco) medium and RPE (retinal pigment epithelial cells) were cultured in DMEM/F-12 (Gibco), all the media containing 10% (v/v) fetal bovine serum (Gibco). All cells were maintained at 37°C with 5% CO_2_.

Parental FRT/TO HeLa cells were used to generate doxycycline-inducible cell lines as described previously (26). Briefly, HeLa FRT/TO cells were transfected with a pcDNA5/FRT/TO vector encoding human AURKA wild-type or K162R (a gift from Stephen Taylor (27), Addgene plasmids 59804 and 59805) together with a plasmid encoding Flp recombinase (pOG44). Stable integrants were then selected with 200 μg/ml hygromycin (InvivoGen) and 4 μg/ml blasticidin (InvivoGen). Transgene expression was induced by treatment with 100 ng/ml doxycycline (Sigma).

When indicated, cells were treated with nocodazole (Selleck Chemicals), etoposide (Selleck Chemicals), thymidine (Sigma), MLN8237 (Selleck Chemicals), VX-689 (Selleck Chemicals), CD532 (Millipore), AurkinA (in house, synthesized from the published structure (23)), AZ3146 (Selleck Chemicals), PHA-767491 (Selleck Chemicals), Simurosertib (MedChem Express), hydroxyurea (Sigma) or DMSO (ThermoFisher Scientific).

### Viability assays

Cell lines were seeded overnight at 5000 cells per well in a 96-well plate in recommended media. Compounds were added the following day and viability was measured at 72 h using the sulforhodamine B (SRB) colorimetric assay as follows: cells were fixed by replacing media with 1% (v/v) trichloroacetic acid (TCA) (Sigma) at 4°C and incubated for 1 h also at 4°C. Wells were washed thoroughly with deionized water before staining with SRB (Sigma) solution (0.057% w/v in 1% v/v acetic acid (VWR)) for 30 min. Stain was aspirated and wells washed with several changes of 1% (v/v) acetic acid until unbound stain was removed, before air-drying. Bound stain was solubilized with 100 μl/well 10 mM Tris (pH 8.0) (Trizma Base, Sigma) with agitation for 10 min. Fluorescence intensity was recorded at Ex540 nm/Em590 nm using a BMG Pherastar Plus plate reader (28).

### Cell cycle analysis

DNA content per cell was measured by flow cytometry using an LSR II (BD Biosciences) flow cytometer. Cells were fixed in 70% ethanol, centrifuged, resuspended in staining buffer (40 μg/ml propidium iodide (PI) (Sigma), 250 μg/ml RNAase A (Sigma), in PBS) and incubated at 37°C in the dark for 30 min before analysis.

In order to calculate the percentage of phospho-MPM-2 positive cells, cells were fixed in 70% ethanol and then washed and resuspended in blocking solution (0.1% Triton X-100 (ThermoFisher Scientific), 1% bovine serum albumin (Sigma) in PBS) for 30 min at room temperature. Then, cells were resuspended in 500 μl antibody solution (anti-phospho-Ser/Thr-Pro MPM-2 Antibody (Millipore, 05-368) in blocking solution), incubated overnight at 4°C, washed twice with blocking solution and resuspend in 500 μl of secondary antibody solution (Alexa Fluor-488 conjugate anti-Mouse IgG secondary antibody (Invitrogen, A-11029) in blocking solution) for 1 h at room temperature. Finally, cells were washed with PBS, resuspended in staining buffer (40 μg/ml PI, 250 μg/ml RNAase A in PBS) and incubated at room temperature in the dark for 30 min before analysis using an LSR II (BD Biosciences) flow cytometer.

For analysis of EdU incorporation, 10 μM EdU (Invitrogen) was added 7 h after cells were released from a mitotic arrest, before S phase entry, and cells were harvested 1 and 2 h after EdU addition. Samples were probed using the Click-iT Plus EdU Flow Cytometry Assay Kit (Invitrogen, C10645) following manufacturers’ instructions and stained with 40 μg/ml PI, 250 μg/ml RNAase before analysis in an LSR II flow cytometer.

### Western blotting

Total protein was isolated by directly lysing the cells in RIPA buffer (50 mM Tris–Cl pH 7.4, 150 mM NaCl, 0.5% sodium deoxycholate (DOC), 0.1% sodium dodecyl sulphate (SDS), 0.1% NP-40) containing 1 mM dithiothreitol (DTT, Sigma), 1 mM phenylmethanesulfonyl fluoride (PMSF, Sigma), EDTA-free Protease Inhibitor Cocktail (Roche), Phosphatase Inhibitors (Cocktail 2 and Cocktail 3, Sigma) and Benzonase (Millipore). Protein lysates were resolved on 4–12% BisTris gels (Invitrogen), transferred onto Immobilon-P, PVDF membrane (Millipore), blocked in tris-buffered saline-0.1% Tween-20 plus 5% non-fat dried milk, and probed with antibodies against ATM (Epitomics 1549-1), pATM(S1981) (Abcam, ab81292), AURKA (Abcam ab52973), β-Actin (Sigma, A5441), CDC45 (Santa Cruz Biotecnology, sc-55569), CDT1 (Santa Cruz Biotecnology, sc-28262), CHK1(Bethyl A300–162A), pCHK1(S345) (Cell Signaling 2348), GAPDH (Proteintech, 60004–1-Ig), H2A.X (Abcam, ab11175), γH2A.X (Cell Signaling 2577), MCM2 (BD Transduction Laboratories, 610700), pMCM2(S53) (Abcam, ab109133), MCM7 (Santa Cruz Biotecnology, sc-9966), MCM10 (Abcam, ab3733), NMYC (Santa Cruz Biotecnology, sc-56729), PCNA (Santa Cruz Biotecnology, sc-25280), PLK1 (Invitrogen 33-1700), POLD1 (Santa Cruz Biotecnology, sc-17776), SLD5 (Abcam, ab121874), TOPBP1 (Abcam, ab2402) or WDHD1 (Abcam, ab72436). Secondary HRP-conjugated antibodies were used and the signal was detected using Amersham enhanced chemiluminescence system (ECL, GE Healthcare).

### Chromatin fractionation

For chromatin fractionation cells were lysed in CSK buffer (10 mM PIPES, pH 7.0, 100 mM NaCl, 300 mM sucrose, 3 mM MgCl_2_) supplemented with 0.7% Triton X-100, EDTA-free Protease Inhibitor Cocktail and Phosphatase Inhibitors (Cocktail 2 and Cocktail 3, Sigma), and centrifuged for 10 min at 13 000 rpm. After centrifugation, the supernatant contained the soluble fraction. The pellet was washed with CSK buffer, resuspended in CSK buffer for sonication and centrifuged for 10 min at 13 000 rpm. After centrifugation, the supernatant contained the chromatin-enriched fraction. Laemmli buffer was added to both fractions and samples were heated at 90°C 5 min. Finally, samples were spun at 13 000 rpm for 1 min before processing by Western blot.

### RNAi and plasmid transfections

DharmaFECT transfection reagent 1 (Dharmacon) was used for RNAi transfections according to manufacturer's instructions. Hs_AURKA_4 FlexiTube RNAi (Qiagen) and AllStars Negative Control RNAi (Qiagen) were used at 25 nM final concentration. Hanks’ Balanced Salt solution (Gibco) was used to dilute the RNAi and the transfection reagent when preparing the transfection mixtures. Transfection reaction mixtures were mixed thoroughly and incubated at room temperature for 10 min before adding dropwise to cells.

Lipofectamine 3000 transfection reagent (ThermoFisher Scientific) was used following manufacturer's instructions for co-transfection of a plasmid encoding Flp recombinase (pOG44) and the modified pcDNA5/FRT/TO vector containing a single amino-terminal Myc tag and the human wild type or K162R AURKA transgenes into HeLa FRT/TO cells. Plasmid concentrations and ratios were used as specified at Flp-In T-REx system (Life Technologies) user manual. Transfection reaction mixtures were mixed thoroughly and incubated at room temperature for 15 min before adding dropwise to cells and culture media was replaced with fresh media after 24 h incubation at 37°C

### Co-immunoprecipitation

HeLa FRT/TO cells were synchronised in G1 by releasing them from a mitotic arrest with nocodazole. Three hours after the release (when cells were in early G1) 2× GI_50_ equivalent concentrations of compounds were added. Cells were then collected 2 h later (before cells enter into S phase), and resuspended in NETN (50 mM Tris⋅Cl, pH 8, 150 mM NaCl, 1 mM EDTA) lysis buffer supplemented with 0.5% NP-40, EDTA-free Protease Inhibitor Cocktail (Roche) and Phosphatase Inhibitors (Cocktail 2 and Cocktail 3, Sigma) for 30 min on ice. Samples were sonicated and centrifuged before collecting the supernatant containing the proteins. In order to immunoprecipitate AURKA, 2 mg protein were incubated with 10μl magnetic beads (Dynabeads^®^, Invitrogen) and 0.5 μg rabbit IgG (Sigma, I5006) for 1 h at room temperature with rotation, in a final volume of 200 μl lysis buffer. Then, the beads were removed and the cleared sample was incubated overnight at 4°C with 20 μl beads previously coated, following manufacturer's instructions, with 6 μg AURKA antibody (Thermofisher, PA1-26351) or 6 μg rabbit IgG as a control. Finally, the beads were washed four times with cold lysis buffer, resuspended in 30 μl 2× Laemmli buffer and heated for 10 min at 70°C. Samples were then incubated for 5 min at 95°C after the addition of 1 μl 1 M β-mercaptoethanol (Sigma). 30 μg protein from the input and the supernatant containing the immunoprecipitated proteins were used for western blot analysis. Membranes were probed against WDHD1 (Abcam, ab72436), MCM7 (Santa Cruz Biotecnology, sc-9966), POLD1 (Santa Cruz Biotecnology, sc-17776) and AURKA (IAK1 anti-Aurora A, BD Biosciences, 610939).

### Mass spectrometry

AURKA was immunoprecipitated from G1 synchronised cell lysates (3 and 5 h after the release from a nocodazole arrest) as described above and the samples containing the co-immunoprecipitated proteins were sent to the Biological Mass Spectrometry and Proteomics Service, at the MRC Laboratory of Molecular Biology, Cambridge, for analysis (Supplementary Table S1). Gene ontology analysis was performed using the Princeton University online service https://go.princeton.edu/cgi-bin/GOTermFinder (29).

## RESULTS AND DISCUSSION

### Allosteric but not catalytic AURKA inhibitors provoke cell cycle arrest in interphase

**Figure 1.**
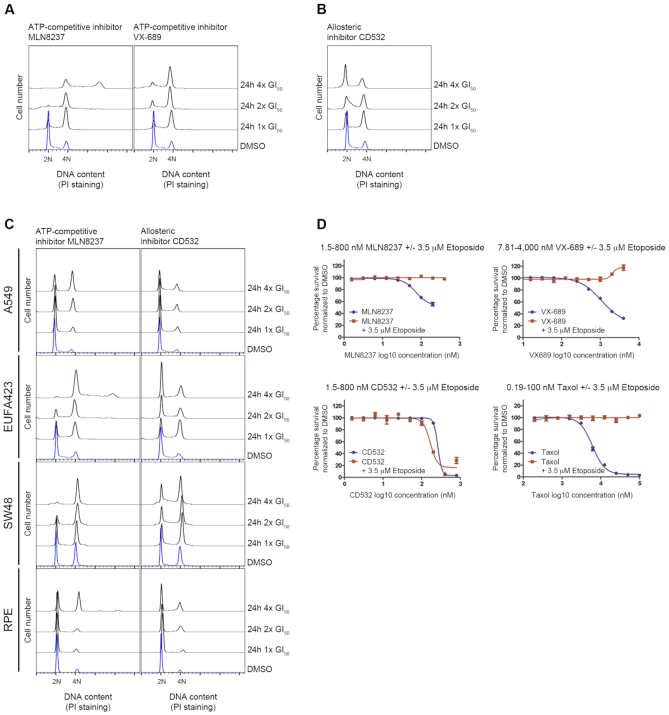
Allosteric but not catalytic AURKA inhibitors provoke cell cycle arrest in interphase. Cell cycle profile of HeLa cells treated with AURKA catalytic inhibitors MLN8237 or VX-689 (**A**), or the allosteric inhibitor CD532 (**B**), at different multiples of GI_50_ equivalent concentrations for 24 h. Plots show cell number on the y-axis, against DNA content measured by propidium iodide (PI) staining, on the x-axis. (**C**) Cell cycle profile of A549, EUFA423, SW48 and RPE cells treated with MLN8237 or CD532 at different GI_50_ equivalent concentrations for 24 h. (**D**) Viability of HeLa cells measured by the SRB assay 72 h after exposure to increasing concentrations of AURKA inhibitors or taxol, without or with 3.5 μM etoposide (equivalent to its GI_25_). Viability as a percentage normalized to control cells exposed to DMSO plotted against the log_10_ concentration in nM of the indicated inhibitor. Values represent the mean ± SEM of three observations. All results are representative of at least three independent repeats.

We first examined the cell cycle profile of cells treated with ATP-competitive (MLN8237 or VX-689) (3,30–33) or allosteric (CD532) inhibitors of AURKA (25,34–38). Gustafson *et al.* have shown that treatment with the allosteric inhibitor CD532 resulted in N-MYC degradation and consequently, a potent loss of S phase entry in N-MYC-amplified neuroblastoma cells, which rely on N-MYC to proliferate. In contrast, AURKA kinase inhibitors (MLN8237 or VX-689) had little or no effect on N-MYC levels and, therefore, resulted in mitotic arrest as a consequence of AURKA catalytic inhibition (25). Surprisingly, we also observed different effects in HeLa cells treated with either class of AURKA inhibitor using a range of equivalent fold GI_50_ concentrations (defined as the dose inducing 50% maximal inhibition of cell proliferation over time) (Figure 1A, B, Supplementary Figure S1A and B), even though these cells express little N-MYC (Supplementary Figure S1C). Catalytic inhibitors arrest the majority of cells in G2-M (Figure 1A), recapitulating features of taxol, a well characterized mitotic inhibitor (Supplementary Figure S1A and D). However, allosteric AURKA inhibitors proposed to alter the structure of the kinase induced only a mild increase in the proportion of cells in G2-M, suggestive of a kinase-independent function during interphase (Figure 1B). A similar phenotype is also observed after AURKA depletion using RNAi (Supplementary Figure S1E), albeit with certain caveats. Cell transfection for AURKA RNAi unavoidably causes some cytotoxicity, increasing the sub-G1 population, and because it takes time to deplete AURKA protein, exerts its effects over a longer duration (24/48 h) than the treatment of cells with AURKAi compounds. Furthermore, we observe similar results in additional cell lines including A549, EUFA423, SW48 or RPE when comparing cell cycle profiles after treatment with catalytic (MLN8237) or allosteric (CD532) inhibitors, despite differences in magnitude consonant with differences in their proliferation rate, indicating that such a non-mitotic, kinase-independent function of AURKA is not cell-type specific (Figure 1C and Supplementary Figure S1F). In these cell lines, N-MYC levels are also virtually undetectable (Supplementary Figure S1C), again suggesting that the G1 arrest observed after CD532 addition is not a consequence of N-MYC proteolysis. Moreover, exposure of cells to a low dose of etoposide sufficient to suppress mitotic entry (Supplementary Figure S1G) sensitized cells to allosteric AURKA inhibitors, but not to catalytic inhibitors or taxol (Figure 1D). Collectively, our findings suggest that allosteric AURKA inhibitors block a distinct non-catalytic function independent of N-MYC during interphase that is not targeted by kinase catalytic inhibitors.

### AURKA is required for the G1-S phase transition

**Figure 2.**
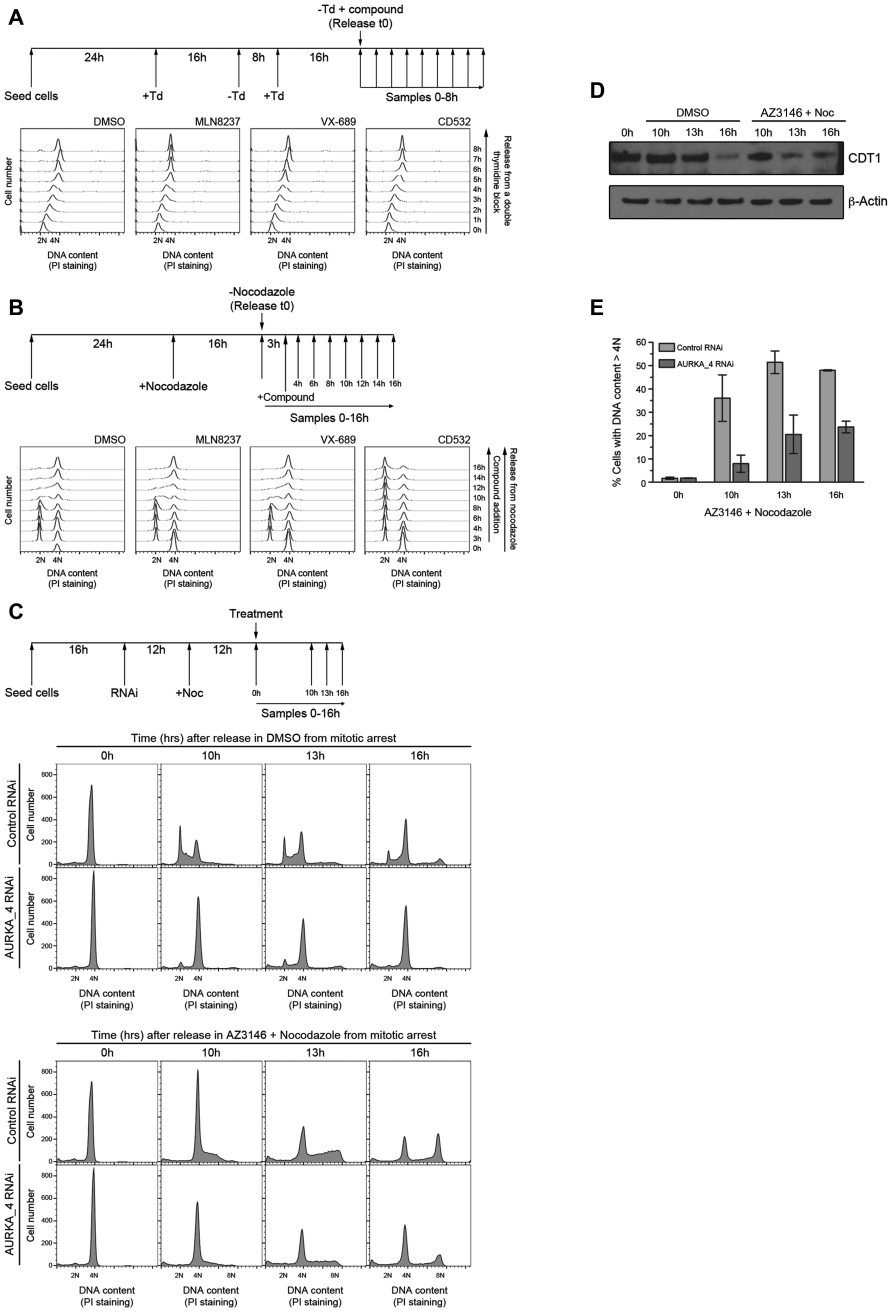
AURKA is required for the G1-S phase transition. (**A**) Upper panel: scheme of the experiment. Cells were synchronized in early S phase by a double thymidine (Td) block (2 mM Td) and AURKA inhibitors at 2× GI_50_-equivalent concentrations were added when cells were released into the cell cycle. Lower panels: cell cycle profile of HeLa cells treated with AURKA inhibitors after release from an early S phase arrest and treated with the inhibitors as indicated in the scheme. Plots show cell number on the y-axis against DNA content measured by propidium iodide (PI) staining on the x-axis. (**B**) Upper panel: scheme of the experiment. Cells were arrested in mitosis by 100 ng/ml nocodazole addition and released into the cell cycle 16 h later. AURKA inhibitors were added at 2× GI_50_-equivalent concentrations when cells were in G1 (3 h after the release from nocodazole). Samples were collected over 16 h. Lower panels: cell cycle profiles of HeLa cells treated as described in the scheme. Cell number (y-axis) was plotted against DNA content (x-axis) measured by propidium iodide (PI) staining. (**C**) Scheme of the experiment (upper panel): cells were transfected with 25 nM negative control or AURKA_4 RNAi and treated with 100 ng/ml nocodazole 12 h after transfection for additional 12 h. AURKA proficient or depleted mitotic arrested cells were then released into the cell cycle in the presence of DMSO or 2 μM AZ3146 plus nocodazole. Lower panels: cell cycle profiles of HeLa cells treated as described in upper panel and released from the mitotic arrest in the presence of DMSO or AZ3146 and nocodazole. (**D**) CDT1 levels detected by western blotting in cells treated as in Figure 2C transfected with 25 nM negative control RNAi. (**E**) Quantification of replicating cells (DNA content >4N after AZ3146 + nocodazole treatment) in the presence or absence of AURKA. Columns represent the mean ± standard deviation (*n* = 3).

Further experiments indicate that this non-catalytic interphase function of AURKA is essential for the G1/S transition, but not for the completion of DNA replication during S phase. Neither catalytic nor allosteric AURKA inhibitors affect S phase progression in cells released synchronously into S phase via a double thymidine (Td) block (Figure 2A). However, when cells synchronized in early G1 phase (3 h after release from a nocodazole-induced mitotic arrest, Figure 2B) are exposed to allosteric but not catalytic AURKA inhibitors, the G1/S transition was impeded (Figure 2B). Consistent with the notion that allosteric inhibitors reveal an interphase function of AURKA, CD532 exerts distinct effects depending on the point of exposure to the compound during the cell cycle. We exposed cells synchronized in mitosis and released into the cell cycle, to a single dose (2× GI_50_) of CD532 or MLN8237, either in early G1 at 3 h after release (Supplementary Figure S2A) or in S phase at 10 h after release (Supplementary Figure S2B). Cell cycle progression was monitored by flow cytometry (Supplementary Figure S2A, S2B). G2/M arrest was validated by increased levels of the mitotic kinase, PLK1 (Supplementary Figure S2C). MLN8237 consistently arrests cells in G2/M, whether added 3 h or 10 h after release (Supplementary Figure S2A and B). By contrast, CD532 induces G2/M arrest when added in S phase (Supplementary Figure S2B, C), but G1 arrest when added at an early time point after release (Supplementary Figure S2A). Moreover, treatment with CD532 in early G1 causes increased CHK1 phosphorylation (Supplementary Figure S2D) suggestive of replication stress, although the absence of functional p53 in HeLa cells discounts checkpoint activation as a cause of G1/S arrest. Collectively, these findings highlight the distinct effects of allosteric versus catalytic AURKA inhibition, and further strengthen our conclusions.

A similar effect to that observed after CD532 addition, that is, a impediment in the G1/S phase transition or sensitization of HeLa cells to a low dose of etoposide sufficient to suppress mitotic entry, also occurs when cells are treated with AurkinA, a small-molecule inhibitor of the AURKA/TPX2 interaction that has been structurally and biochemically validated but whose cellular potency is under optimization ((23), Supplemental Figure S2E–I). That two allosteric inhibitors with significantly different chemical structures reveal such a function suggests that it does not arise from off-target effects.

Indeed, genetic experiments provide further evidence supporting that such a function does not arise from off-target effects. Experiments to test the function of AURKA during the G1/S transition proved technically challenging. AURKA is essential for the completion of mitosis, and currently available genetic methods preclude its timed depletion after exit from mitosis. Therefore, we devised a procedure to drive cells depleted of AURKA during the M phase into G1. To this end, we first depleted AURKA using RNAi in cells arrested in mitosis by nocodazole treatment (Figure 2C and Supplementary Figure S2J), before forcing mitotic exit with AZ3146, an MPS1 inhibitor that overrides the spindle assembly checkpoint and permits AURKA-depleted cells to complete mitosis and enter G1 ((39), ‘Release in DMSO’ panels in Figure 2C versus Supplementary Figure S2K). In order to analyze the implication of AURKA in the G1/S transition, nocodazole exposure was maintained during AZ3146 treatment to prevent cell division, and thereby circumvent asynchrony in mitotic timing (‘Release in AZ3146 + nocodazole’ panels in Figure 2C). Hence, in these experiments, cells move from mitosis into G1 and S phase duplicating the genome in the absence of cell division, thereby increasing their DNA content from 4N to 8N. These findings cannot simply be attributed to DNA re-replication after prolonged mitotic arrest, because CDT1 levels fall when cells synchronized with nocodazole are treated with AZ3146 to bypass the spindle assembly checkpoint and re-initiate DNA replication (Figure 2D). Instead, these findings imply that our approach enables licensing of new origins in a subsequent cell cycle. Whereas AURKA-expressing control RNAi-treated cells initiate DNA replication to enter S phase (‘Release in AZ3146 + nocodazole’ upper panels in Figure 2C), AURKA-depleted cells show an impediment to DNA replication (‘Release in AZ3146 + nocodazole’ lower panels in Figure 2C and E). Taken together, our results suggest that AURKA performs a kinase-independent function that is essential for the G1/S phase transition, but dispensable for replication progression once DNA synthesis has initiated.

### AURKA performs a function in DNA replication independent of its kinase activity

**Figure 3.**
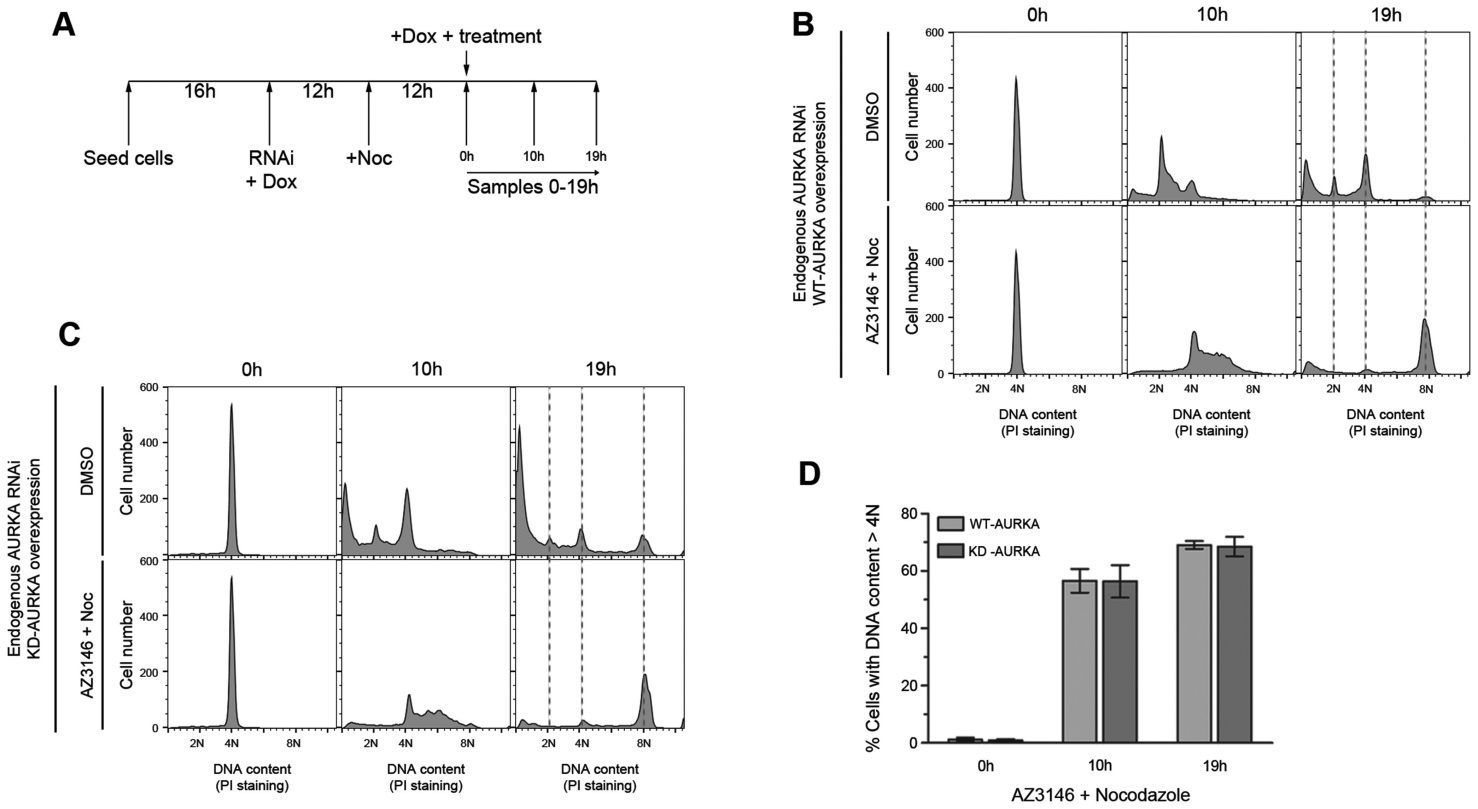
AURKA performs a function in DNA replication independent of its kinase activity. (**A**) Scheme of the experiment: doxycycline-inducible FRT/TO HeLa cells harboring a wild type or a kinase dead Myc-tagged AURKA transgen were transfected with 25 nM negative control or AURKA_4 RNAi (specific for endogenous AURKA) in media containing 100 ng/ml doxycycline to induce overexpression of wild type- or kinase dead-AURKA protein. 12 h after the transfection cells were treated with 100 ng/ml nocodazole for additional 12 h. Endogenous-AURKA proficient or depleted mitotic arrested cells were then released into the cell cycle in the presence of DMSO or 2 μM AZ3146 plus nocodazole, in media containing doxycycline. Cell cycle profiles of endogenous-AURKA depleted HeLa cells treated as described in A and overexpressing a wild type (**B**) or a kinase dead (**C**) version of AURKA. (**D**) Quantification of replicating cells (DNA content >4N after AZ3146 + nocodazole treatment) expressing either a wildtype or a kinase-dead form of AURKA in the absence of endogenous AURKA. Columns represent the mean ± standard deviation (*n* = 2). All figures are representative of at least two independent experiments.

To verify that the role of AURKA revealed by allosteric inhibitors was independent of its catalytic activity, we next tested if the failure of AURKA-depleted cells to transit from G1 to S phase can be prevented by the expression of a wild-type AURKA, as well as by a catalytically-inactive AURKA protein. To this end, FRT/TO HeLa cells expressing a doxycycline-inducible wild-type or kinase-dead Myc-tagged AURKA transgene were depleted of endogenous AURKA by RNAi in the presence of doxycycline. Cells treated in this way, which are expected to express either the wild type- or kinase-dead AURKA protein, were synchronously released into cell cycle from mitotic arrest, as before by exposure to AZ3146 plus nocodazole, alongside DMSO-treated controls (Figure 3A, Supplementary Figure S3A). As expected, reconstitution with a wild type form of AURKA permits cells to progress both through S phase and mitosis (Figure 3B). However, reconstitution with a catalytically-inactive form of AURKA suffices to overcome this block in the G1/S phase transition (‘AZ3146 + nocodazole’ panels in Figure 3C and D) but, consistent with the widely established kinase-dependent role of AURKA in mitosis, fails to support mitotic cell division, inducing cell death at the late time-points (‘DMSO’ panels in Figure 3C versus Supplementary Figure S3B). Collectively, these findings provide genetic evidence that a kinase-independent function of AURKA—distinct from its putative role during mitosis in supporting geminin stability (10)—is essential for the G1/S transition.

### AURKA has a role in the activation of replication origins independent of its kinase activity

Our findings prompted us to examine the role of AURKA in the mechanisms that initiate DNA replication. Several events crucial in ensuring that DNA replication occurs only once per cell cycle take place before entry into S phase. First, an inactive form of the hexameric MCM2–7 helicase assembles with the CDT1 factor on chromatin at sites that mark origins of replication to form the pre-replicative complex (‘licensing’). Pre-replicative complex assembly occurs within a specific time window between late mitosis and early G1, when cyclin-dependent kinase (CDK) activity is low, and the activity of the anaphase-promoting complex (APC/C) is high. Subsequently, pre-replicative complexes are activated to initiate DNA replication (‘firing’) by the sequential chromatin recruitment of proteins such as CDC45 and TOPBP1, followed by factors like WDHD1, MCM10 and PCNA among others, collectively serving to induce the formation of the replicative polymerase holoenzyme. Origin firing occurs only after APC/C activity has been extinguished whereas CDK activity reaccumulates, triggering the G1/S transition (40,41).

**Figure 4.**
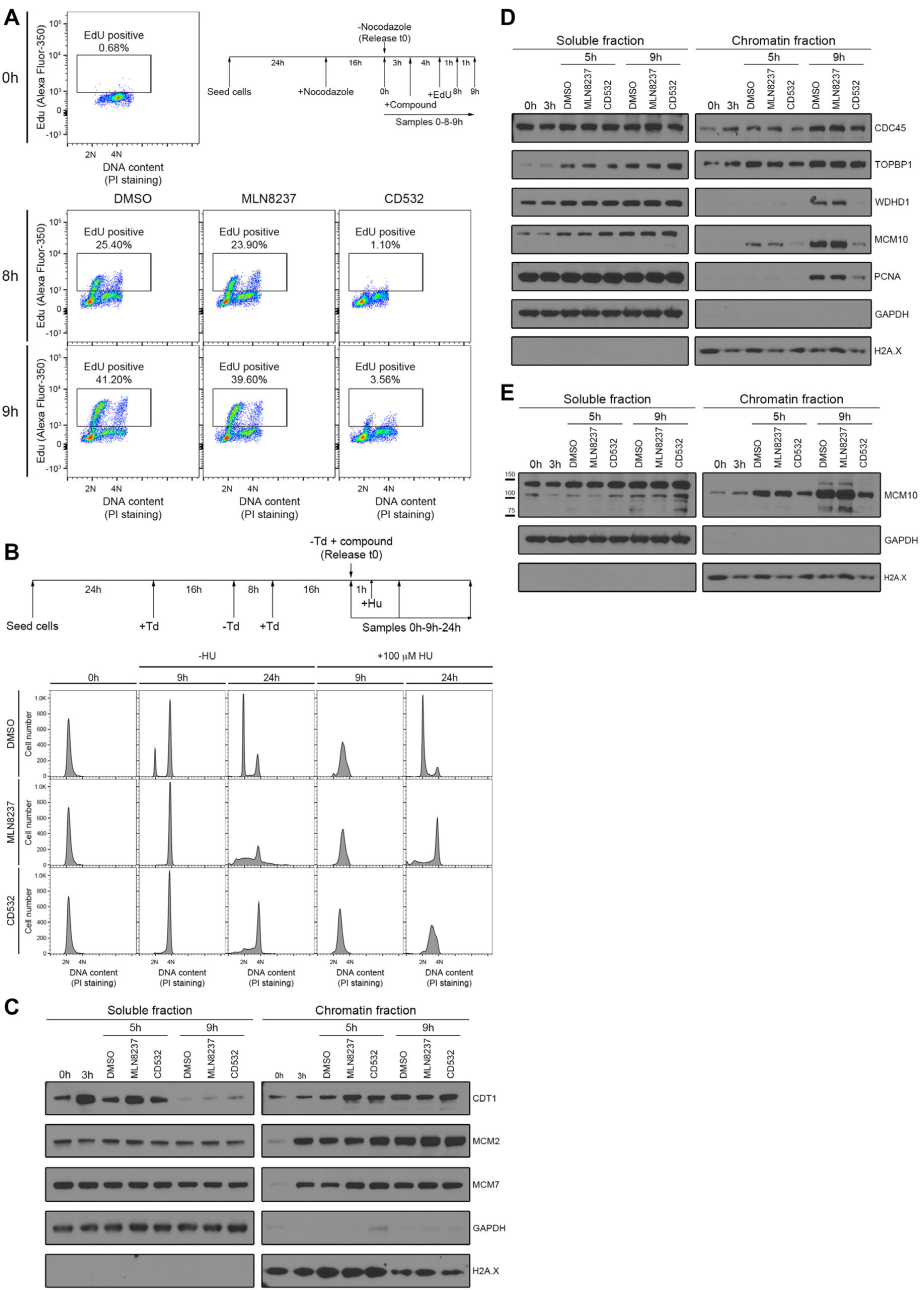
AURKA has a role in the activation of replication origins independent of its kinase activity. (**A**) EdU incorporation analyses in cells released into G1 from a nocodazole-induced mitotic arrest. 2× GI_50_ equivalent concentrations of AURKA inhibitors were added 3 h after the release from nocodazole, when cells were in G1; 10 μM EdU was added 7 h after the release from the mitotic arrest, before cells entered into S phase. Plots show Edu content measured by Alexa Fluor-350 intensity on the y-axis, against DNA content measured by propidium iodide (PI) staining, on the x-axis. The percentage of replicating EdU positive cells is shown over the box enclosing the positive cells. (**B**) Scheme of the experiment (upper panel): cells were arrested in early S phase by a double thymidine treatment and then released from the block in the presence of AURKA inhibitors at 2× GI_50_ equivalent concentrations. One hour later, when cells were progressing through S phase, the firing of new origins was forced by addition of a low dose of hydroxyurea (100 μM) and samples were collected over the time. Lower panels: cell cycle profiles of HeLa cells treated as described above. Cell number (y-axis) was plotted against DNA content (x-axis) measured by propidium iodide (PI) staining. (**C**) Protein levels at the soluble and chromatin fractions of HeLa cells arrested in mitosis by nocodazole addition and then release into the cell cycle. 2× GI_50_ equivalent concentrations of AURKA inhibitors were added when cells were in G1 (3 h after the release) and samples were collected at the indicated times. After lysis, the soluble and chromatin fractions were separated and immunoblotted for the indicated proteins involved in the licensing of the replication origins. (**D**) HeLa cells were treated as in *C* and the soluble and chromatin fractions were separated and immunoblotted for the indicated proteins involved in the firing of the replication origins. (**E**) Overexposed membrane probed against MCM10 antibody shown in D. All results are representative of at least three independent repeats.

We find that allosteric but not catalytic AURKA inhibitors suppress DNA replication initiation during the G1/S transition (Figure 2B, Supplementary Figure S2A). Thus, the incorporation into nascent DNA of the nucleotide analogue EdU in cells transiting from G1 to S phase is inhibited by the allosteric inhibitor CD532, but not the catalytic inhibitor MLN8237 (Figure 4A). Moreover, we have already shown that AURKA is dispensable for progression through S phase, since the effect of the allosteric inhibitors on replication is only observed when cells are treated in G1, before the initiation of DNA duplication has taken place (Figure 2A versus B, compare Supplementary Figure S2A versus B), consistent with the fact that the majority of the origins required to complete DNA replication during an unperturbed S phase are activated during the G1/S transition.

The vast majority of origins licensed during the G1/S transition remain dormant during an unperturbed S phase, but may be activated later during S phase should cells encounter lesions that prevent or impede replication fork progression (42). To address whether AURKA was necessary for the activation of these late origins, we arrested cells using a double thymidine block and then released them synchronously into S phase. One hour later, during S phase progression, we exposed the synchronized cells to a low dose of the replication inhibitor hydroxyurea (Figure 4B), which is reported to trigger the activation of dormant origins to overcome replication inhibition and complete genome duplication (42,43). Whereas inhibition of AURKA kinase catalytic activity had no effect in S phase execution and cells only arrest when they reach mitosis (MLN8237 treatment in Figure 4B), we observe that an allosteric inhibitor of AURKA prevented the completion of DNA replication, but only in the presence of low doses of hydroxyurea (CD532 treatment in Figure 4B), when new origin activation is necessary (42,43). Collectively, our results therefore suggest that AURKA is essential not only for DNA replication firing during the G1/S transition, but also for the firing of dormant, licensed origins when cells encounter replication stress later during S phase.

Consistent with these observations, neither catalytic nor allosteric AURKA inhibitors affect the chromatin loading of CDT1, MCM2 or MCM7 (Figure 4C), suggesting that AURKA is dispensable during G1 for pre-replicative complex assembly and replication licensing, in contrast to a prior report (10). Similarly, both types of AURKA inhibitors do not affect the phosphorylation of MCM2 (Supplementary Figure S4A) or the loading of CDC45 or TOPBP1 (Figure 4D), suggesting that the early events during replication firing proceed normally. Remarkably however, the allosteric AURKA inhibitor CD532—but not the catalytic inhibitor MLN8237—markedly diminished the loading of WDHD1, MCM10, PCNA (Figure 4D). Moreover, chromatin loading of SLD5, a component of the GINS complex necessary for replisome firing, was also impaired (Supplementary Figure S4B). Interestingly, the observed defect in MCM10 loading is evident 5 hrs after release from a nocodazole arrest even when CD532-treated cells remain in G1 (as marked by the absence of PCNA on chromatin, Figure 4D). No detectable effect on the level of chromatin-bound AURKA was observed for either CD532 or MLN8237 (Supplementary Figure S4C). Thus, our findings demonstrate that a kinase-independent function of AURKA is required for DNA replication firing by promoting the chromatin loading of proteins required for activation of the replicative polymerase.

Interestingly, the chromatin loading of TOPBP1 remains unaffected (Figure 4D). Thus, our results suggest that the point at which a novel non-catalytic function of AURKA is required for replication initiation falls between TOPBP1 recruitment and GINS assembly. These results not only speak against the notion that the requirement for AURKA in G1 is somehow a consequence of prolonged mitosis or replication fork instability, but also better define a novel non-catalytic function of AURKA during the temporal progression of replisome assembly that is distinct from the role of AURKA kinase activity in replication fork protection during S phase shown previously (12).

Budding yeast Mcm10 is di-ubiquitinated during S phase, permitting recognition by PCNA (44). However, it remains unclear whether human MCM10 undergoes similar modifications, and whether such modifications regulate its function, even though two forms of human MCM10 whose gel migration corresponds to mono- and diubiquitinated species can be detected in late G1 and through S phase (45,46). In G1-synchronised cells (5 h after nocodazole release), a slow-migrating form of MCM10 suggestive of a di-ubiquitinated species is not detected on chromatin, consistent with the absence of PCNA recruitment on chromatin before S phase initiation (Figure 4D and E). When cells initiate replication (9 hrs after nocodazole release), this slow-migrating MCM10 form can be detected in the nuclear-soluble, but not chromatin-bound, fraction of CD532-treated cell extracts (Figure 4E). Moreover, CD532 exposure decreases the chromatin-bound level of the faster-migrating MCM10 form and markedly reduces the level of the slow-migrating form of MCM10, compared to AURKA catalytic inhibitor or control DMSO exposure. These findings suggest that CD532 suppresses the loading of MCM10 onto chromatin, which in turn diminishes PCNA recruitment, to inhibit replication origin firing.

Interestingly, mass spectometry data showed that AURKA forms a macromolecular complex with several proteins involved in DNA replication initiation (Supplementary Table S1): it co-immunoprecipitates with MCM7, WDHD1 and POLD1 in an extract prepared from cells synchronized in G1. However, complex formation is not affected by either catalytic or allosteric AURKA inhibitors (Supplementary Figure S4D). Thus, these results suggest that chromatin loading of factors involved in replication firing requires an AURKA function that is blocked by allosteric but not catalytic inhibitors; this function is, however, dispensable for complex formation.

### AURKA allosteric inhibitors and DDK inhibitors mutually cross-sensitize HeLa cells

**Figure 5.**
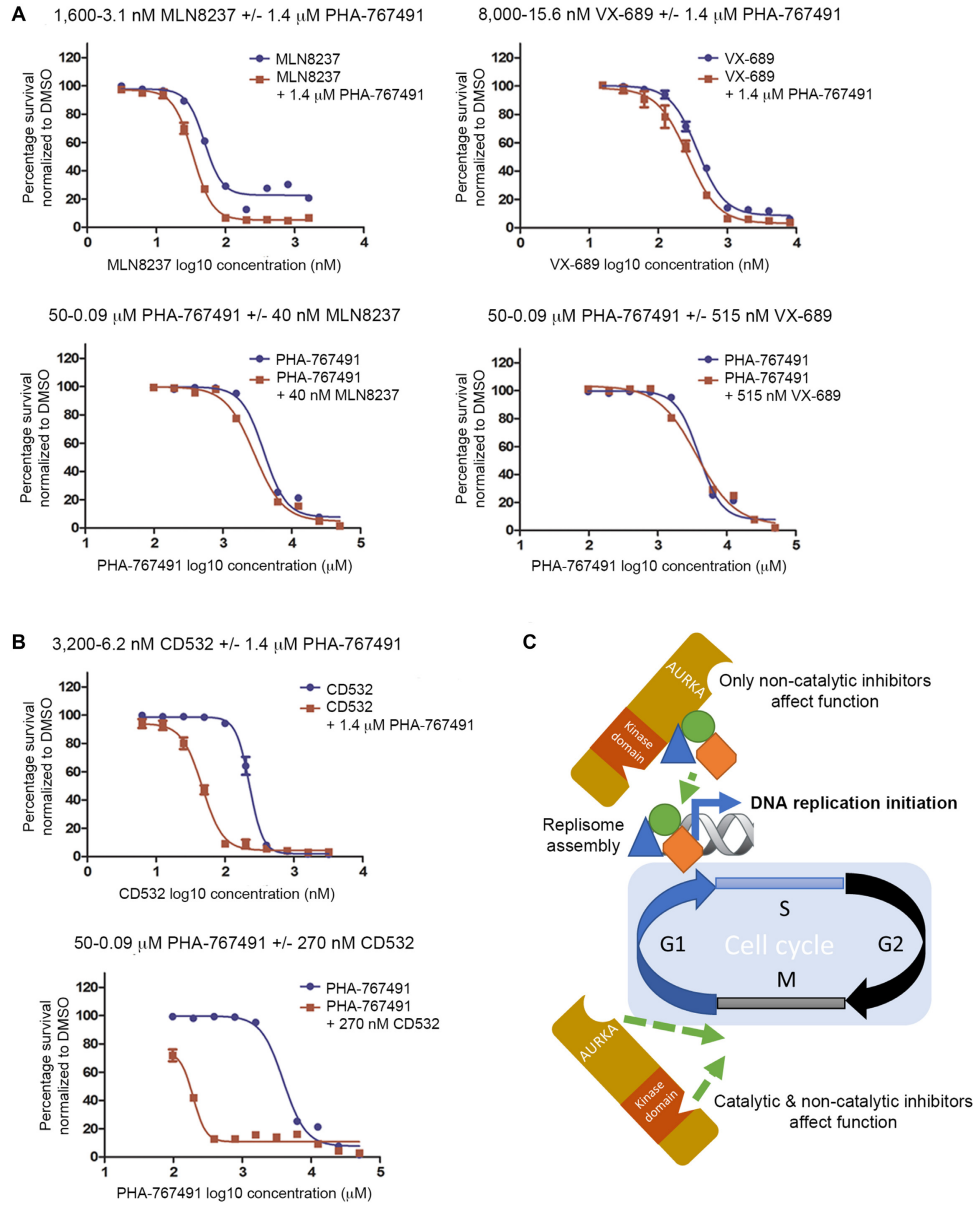
AURKA allosteric inhibitors and DDK inhibitors mutually cross-sensitize HeLa cells. (**A**) Cell viability of HeLa cells treated with decreasing concentrations of MLN8237 or VX-689 without or with 1.4 μM PHA-767491 (equivalent to its GI_25_) (upper panels), or decreasing concentrations of PHA-767491 without or with 40 nM MLN8237 or 515 nM VX-689 (equivalent to its GI_25_) (lower panels). Viability was measured by the SRB assay and represented as a percentage normalized to control cells exposed to DMSO, plotted against the log_10_ concentration of the indicated inhibitor. Values represent the mean ± SEM of three observations. (**B**) Cell viability of HeLa cells treated with combinations of CD532 together with PHA-767491 as indicated in A. All results are representative of at least three independent repeats. (**C**) Diagram depicting kinase dependent and independent functions of AURKA in mitosis and DNA replication respectively (see text for details).

Our observation that a kinase-independent function of AURKA is essential for the initiation of DNA replication prompted us to test whether dividing cells would be sensitized to the effects of an allosteric AURKA inhibitor in combination with an inhibitor of replication initiation. To this end, we tested the viability of cells exposed to PHA-767491, a CDC7 inhibitor that blocks the Dbf4-dependent kinase essential for DNA replication (47), in combination with different types of AURKA inhibitors. While the combination of catalytic AURKA inhibitors with PHA-767491 had no additive effect (Figure 5A and Supplementary Figure S5A), exposure to the allosteric AURKA inhibitors CD532 or AurkinA cross-sensitized HeLa cells to the cytotoxic effect of PHA-767491 (Figure 5B, Supplementary Figure S5B, C). Similar results obtained when cells were treated with AURKA inhibitors in combination with Simurosertib, an alternative CDC7 inhibitor (48), further confirm this cross-sensitization (Supplementary Figure S5D, E). These findings raise the possibility that such a drug combination might exhibit enhanced efficacy in cancer therapy.

### Implications

The findings we report here identify a novel function of human AURKA in the initiation of DNA replication. AURKA operates after pre-replicative complex assembly and early steps in replication firing to promote the chromatin loading of essential factors for replicative polymerase activation. This AURKA function is independent of its kinase catalytic activity and, accordingly, can be blocked by a new class of allosteric inhibitors but not by classical catalytic-site inhibitors, causing cell cycle arrest at the G1/S phase transition (Figure 5C). By contrast, the canonical, kinase-dependent role of AURKA in mitosis can be blocked by both catalytic and allosteric inhibitors (Figure 5C). Moreover, this function is also required for the reactivation of dormant replication origins following DNA damage during S phase. Thus collectively, our findings define an AURKA-dependent step required for replicative polymerase activation via the chromatin loading of SLD5, WDHD1, MCM10 and PCNA, providing fresh insights into the mechanisms that control human DNA replication. We speculate that the frequent overexpression of AURKA in many different forms of human cancer may serve to dysregulate this replicative function, promoting its role as an oncogenic driver. Interestingly, allosteric AURKA inhibitors that disrupt its replicative function effectively cross-sensitize cancer cells to inhibitors of the CDC7 replicative kinase under clinical development, suggesting a new approach with potential value in the combination therapy of human cancers. Thus, our work demonstrating a novel non-mitotic function of AURKA offers fresh biologic insights as well as potential therapeutic impact.

## Supplementary Material

gkaa570_Supplemental_Files
