# Supplementary material for: A kinase-independent function for AURORA-A in replisome assembly during DNA replication initiation
Source: Nucleic Acids Res. 2020 Jul 11;48(14):7844–55. doi: 10.1093/nar/gkaa570 (PMC7430631; doi:10.1093/nar/gkaa570)
Supplement: gkaa570_Supplemental_Files [file gkaa570_supplemental_files.zip › Supplementary Data.pdf]

A kinase-independent function for AURORA-A in replisome assembly during DNA replication initiation

Estrella Guarino Almeida, Xavier Renaudin & Ashok R. Venkitaraman

Supplementary data

Supplementary Figure S1. Related to Figure 1. (**A**) HeLa cells were treated with increasing concentrations of AURKA inhibitors or taxol for 72 hrs before cell viability was determined using the SRB assay normalized to DMSO controls and GI_{50} was calculated. (**B**) Cell cycle profile of HeLa cells treated with AURKA catalytic inhibitors (MLN8237 or VX-689) or the allosteric inhibitor CD532 at a GI_{50} equivalent concentration for the indicated times. (**C**) N-MYC levels deterected by Western blotting in N-MYC non-amplified (HeLa, A549, EUFA423, SW48 and RPE) and N-MYC-amplified (SK-N-BE(2)) cell lines. (**D**) Cell cycle profiles of HeLa cells treated with taxol at a GI_{50} equivalent concentration for the indicated times (upper panel) or at the indicated multiples of GI_{50} equivalent concentrations for 24 hrs (lower panel). (**E**) Cell cycle distribution of HeLa cells 24, 48 or 72 hrs after transfection with 25 nM negative control or AURKA_4 RNAi (upper panel), and Western blot analysis of AURKA levels at the same time points after RNAi transfection (lower panel). (**F**) GI_{50} values calculated for A549, EUFA423, SW48 or RPE cells after treatment with increasing concentrations of MLN8237 or CD532 for 72 h before cell viability was determined using the SRB assay normalized to DMSO controls. (**G**) Cell cycle analysis of HeLa cells after 24 hrs treatment with the 4x GI_{50} concentration of AURKA inhibitors or Taxol in combination with etoposide at GI_{25} (3.5μM). The percentage of phospho-MPM2 positive cells in G2/M phases is shown in the right corner. All results are representative of at least two independent repeats.

Supplementary Figure S2. Related to Figure 2. (**A**) Upper panel: scheme of the experiment. Cells were arrested in mitosis by 100 ng/ml nocodazole addition and released into the cell cycle 16 hrs later. AURKA inhibitors at 2x GI_{50} equivalent concentrations were added when cells were in G1 (3 hrs after the release from nocodazole). Samples were collected over 15 hrs. Lower panels: cell cycle profiles of HeLa cells treated as described in the scheme. Cell number (y-axis) was plotted against DNA content (x-axis) measured by propidium iodide (PI) staining. (**B**)

Upper panel: scheme of the experiment. Cells were arrested in mitosis by nocodazole addition and released into the cell cycle 16 hrs later. 2x GI_{50} equivalent concentrations of AURKA inhibitors were added when cells were in S phase (10 hrs after the release from nocodazole). Samples were collected over 24 hrs. Lower panels: cell cycle profiles of HeLa cells treated as described in the scheme. Cell number (y-axis) was plotted against DNA content (x-axis) measured by propidium iodide (PI) staining. (**C**) Western blot analysis of PLK1 levels in cells treated as in indicated in *B*. (**D**) Cells were treated as specified in *A*. Samples were collected over 15 hrs and indicated protein lelvels were analyzed by Western blot. (**E**) Cell cycle profile of HeLa cells treated with AURKA allosteric inhibitor AurkinA at a GI_{50} equivalent concentration for the indicated times (left panels), or at different multiples of GI_{50} equivalent concentrations for 24 hrs (right panels). AurkinA GI_{50} (119.9 μM) was calclulated from viability curves generated by the SRB assay as specified in Figure 1. (**F**) Viability of HeLa cells measured by the SRB assay 72 hrs after exposure to increasing concentrations of AurkinA without or with 3.5μM etoposide (equivalent to its GI_{25}). Viability is shown as a percentage normalized to control cells exposed to DMSO, plotted against AurkinA log10 concentration in μM. Values represent the mean ± SEM of three observations. (**G**) Cell cycle analysis of HeLa cells after 24 hrs treatment with the 4x GI_{50} concentration of AurkinA in combination with etoposide at GI_{25}. The percentage of phospho-MPM2 positive cells in G2/M phases is shown in the right corner. (**H**) Cell cycle profile of HeLa cells treated with AurkinA after release into early S phase after a bouble thymidine block, as specified in Figure 2A. (**I**) Cell cycle profiles of HeLa cells released from a nocodazole arrest and treated with AurkinA in G1, as indicated in Figure 2B. (**J**) AURKA levels in HeLa cells transfected with 25 nM negative control or AURKA_4 RNAi and treated with 100 ng/ml nocodazole 12 hrs after transfection for additional 12 hrs. AURKA proficient or depleted mitotic arrested cells were then released into the cell cycle in the presence of DMSO,

AZ3146 or AZ3146 plus nocodazole. Otherwise indicated, the Western blot shows protein levels 16 hrs after release. (**K**) Cell cycle profiles of HeLa cells treated as described in Figure 2C and release from the mitotic arrest in the presence of AZ3146.

Supplementary Figure S3. Related to Figure 3. (**A**) AURKA levels in doxycycline-inducible FRT/TO HeLa cells harboring a wild type or a kinase dead Myc-tagged AURKA transgen transfected with 25 nM negative control or AURKA_4 RNAi (specific for endogenous AURKA) in media containing 100 ng/ml doxycycline to induce overexpression of wild type or kinase dead Myc-tagged AURKA protein. 12 hrs after the transfection cells were treated with 100 ng/ml nocodazole for additional 12 hrs. Endogenous-AURKA proficient or depleted mitotic arrested cells were then released into the cell cycle in the presence of DMSO or 2 μM AZ3146 plus nocodazole, in media containing doxycycline and samples were collected 19 hors after release, unless otherwise indicated. (**B**) Cell cycle profiles of FRT/TO HeLa cells harboring a wild type or a kinase dead Myc-tagged AURKA transgen, transfected with 25 nM negative control RNAi, and treated as described in Figure 3A to overexpress a wild type or a kinase dead type of Myc-tagged AURKA. All results are representative of at least two independent repeats.

Supplementary Figure S4. Related to Figure 4. (**A**) Cells were arrested in mitosis by 100 ng/ml nocodazole addition and released into the cell cycle 16 hrs later. 2x GI_{50} equivalent concentrations of AURKA inhibitors were added when cells were in G1 (3 hrs after the release from nocodazole). Samples were collected at the indicated time points and MCM2 and phospho-MCM2 levels were analysed by western blot. (**B**) SLD5 levels at the soluble and chromatin fractions of HeLa cells arrested in mitosis by nocodazole addition and then release into the cell cycle. AURKA inhibitors were added at 2x GI_{50} equivalent concentrations when cells

were in G1 (3 hrs after the release) and samples were collected at the indicated times. After lysis, the soluble and chromatin fractions were separated and immunoblotted for the indicated proteins. (**C**) AURKA levels in soluble and chromatin fractions of G1 cells released from a nocodazole-induced mitotic arrest and treated with AURKA inhibitors, as specified in *B*. (**D**) Western blot analysis of replication proteins detected by mass spectrometry that co-immunoprecipitate with AURKA in G1 cells. HeLa cells were arrested in mitosis by nocodazole addition and then release into the cell cycle. AURKA inhibitors were added at 2x GI_{50} equivalent concentrations when cells were in G1 (3 h after the release) and samples were collected 2 hours after compound addition. Total levels of the indicated proteins were analyzed by Western blot. The figures are representative of at least two independent repeats.

Supplementary Figure S5. Related to Figure 4. (**A**) & (**B**) GI_{50} calculated from viability curves shown in Figure 5A and 5B. Fold difference on each pair of combinations is shown on the right colum of the table. (**C**) Cell viability of HeLa cells treated with decreasing concentrations of AurkinA without or with 1.4 µM PHA-767491 (equivalent to its GI_{25}) (left panels), or decreasing concentrations of PHA-767491 without ot with 90 µM AurkinA (equivalent to its GI_{25}) (right panels). (**D**) Cell viability of HeLa cells treated with decreasing concentrations of MLN8237 or VX-689 without or with 3.2 µM Simurosertib (equivalent to its GI_{25}) (upper panels), or decreasing concentrations of Simurosertib without ot with 40 nM MLN8237 or 515 nM VX-689 (equivalent to its GI_{25}) (lower panels). (**E**) Cell viability of HeLa cells treated with combinations of CD532 together with Simurosertib as indicated in *C*. In *C*, *D* and *E*, viability was measured by the SRB assay and represented as a percentage normalized to control cells exposed to DMSO, plotted against the log_{10} concentration of the indicated inhibitor. Values represent the mean ± SEM of three observations. The tables show GI_{50} calculated from viability curves and fold

difference on each pair of combinations is indicated on the right colum of the table. All results are representative of at least three independent repeats.

Supplementary Table S1. AURKA was immunoprecipitated from G1 synchronised cell lysates (3 and 5 hours after the release from a nocodazole arrest) and the samples containing the co-immunoprecipitated proteins were analysed by mass spectrometry as indicated in the main text. Gene ontology analysis was performed using the Princeton University online service https://go.princeton.edu/cgi-bin/GOTermFinder.

Supplementary Figure S1

A

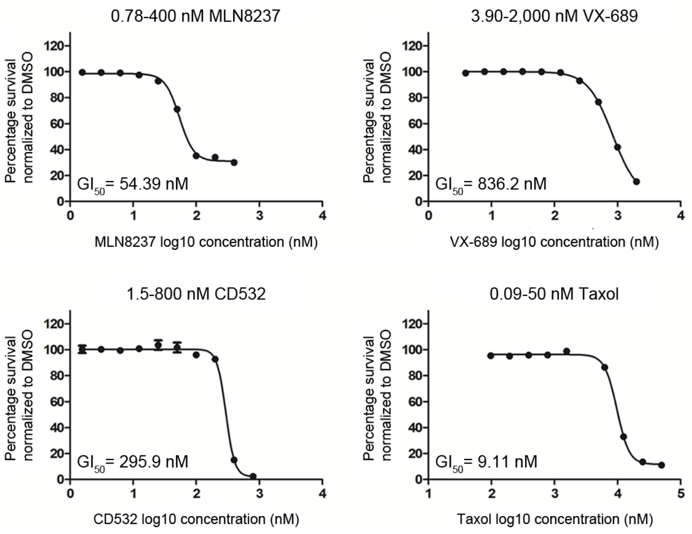

B

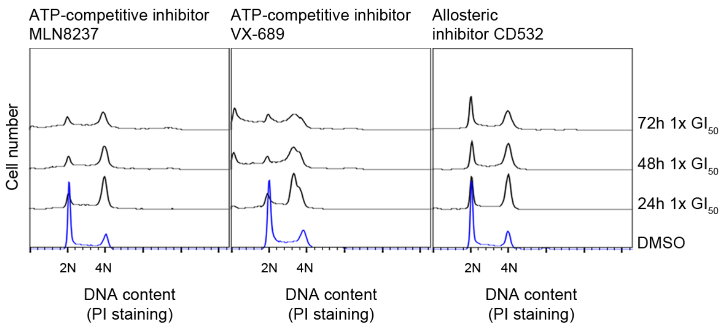

C

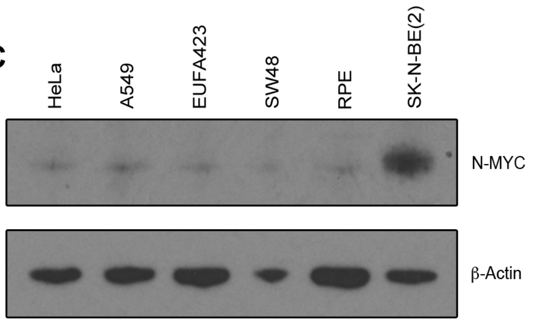

E

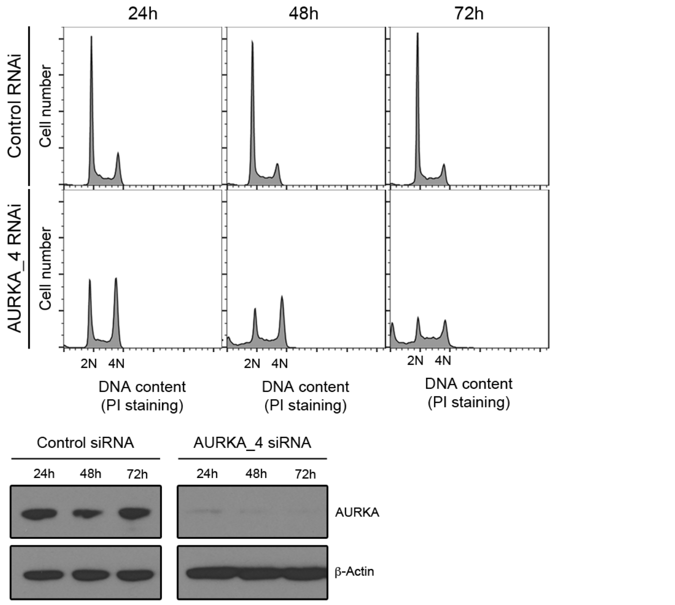

D

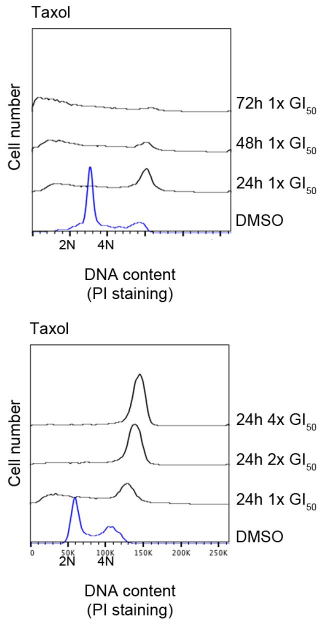

G

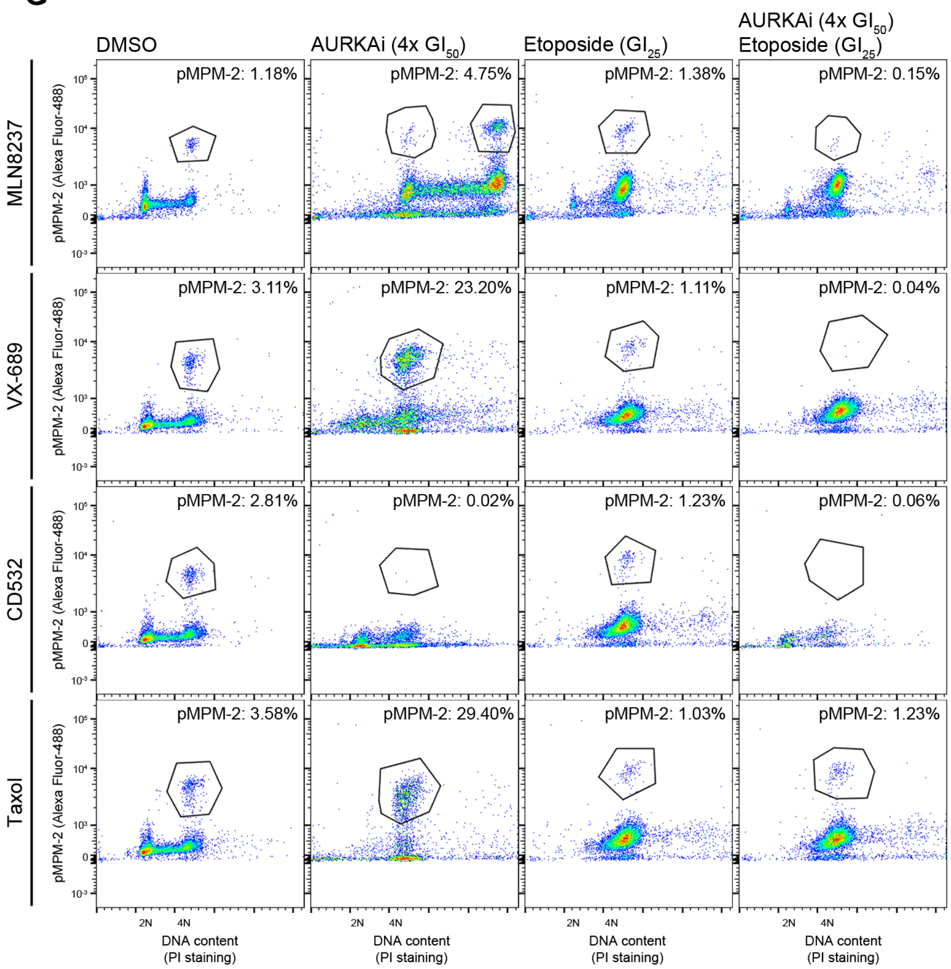

F

GI50	**MLN8237**	**CD532**
**A549**	75 nM	1 µM
**SW48**	8 nM	25 nM
**RPE**	25 nM	1 µM
**EUFA423**	75 nM	285 nM

Supplementary Figure S2

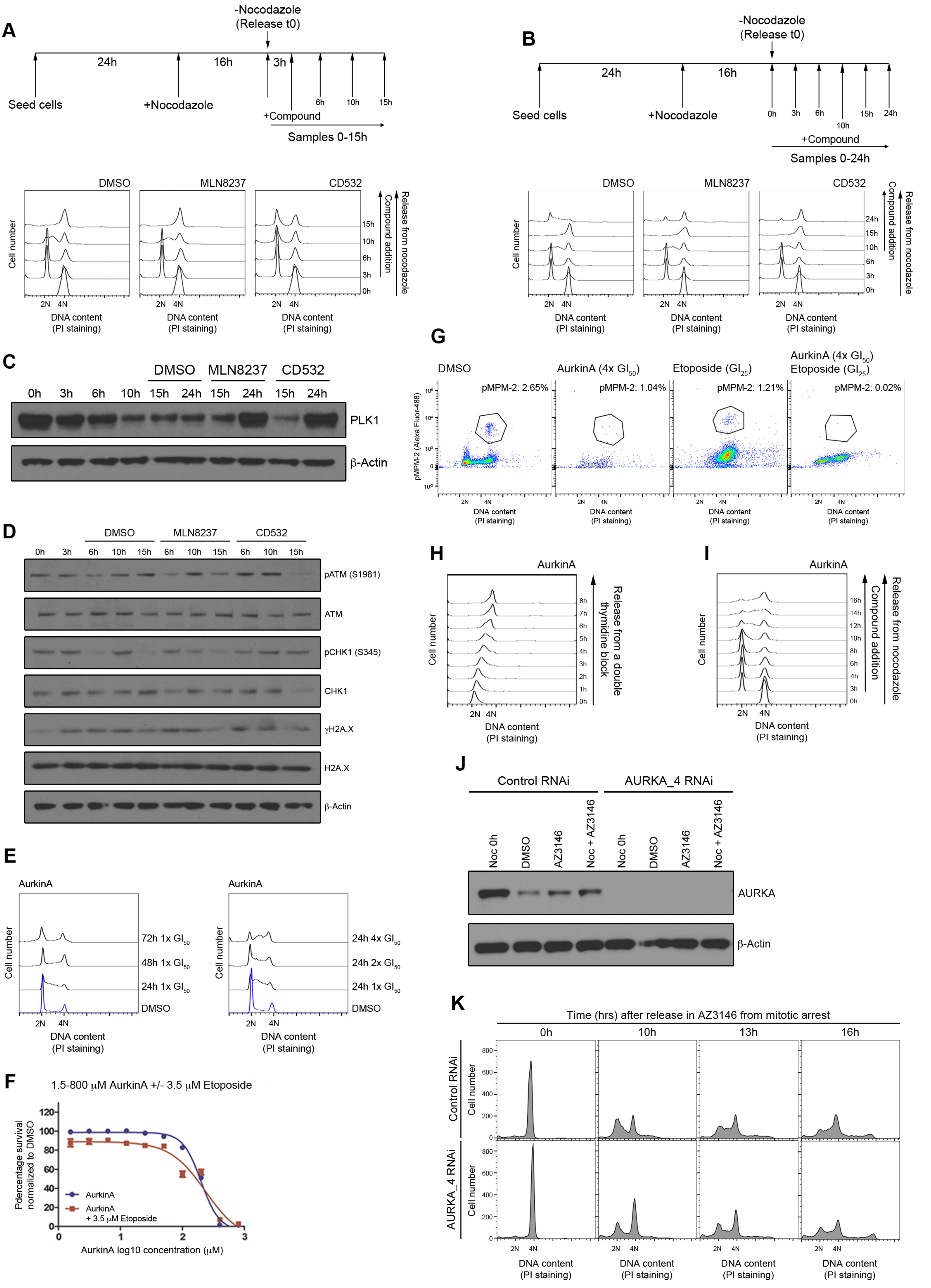

Supplementary Figure S3

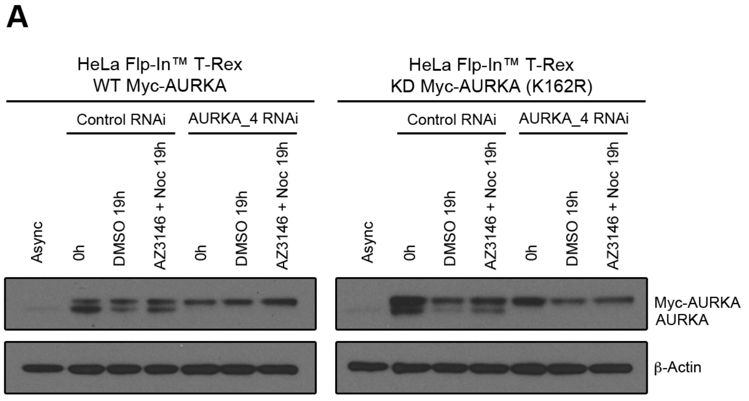

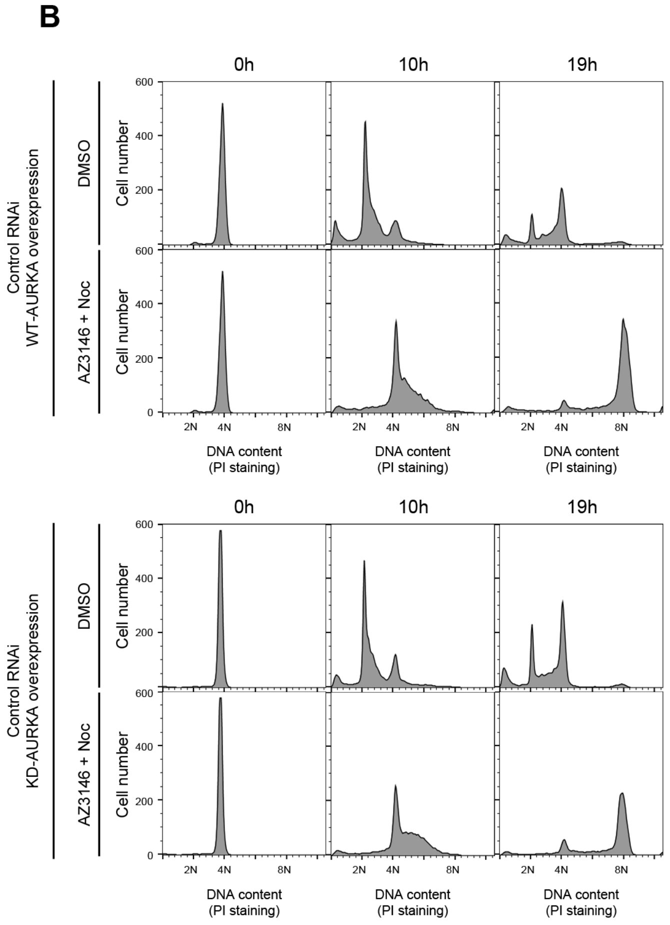

Supplementary Figure S4

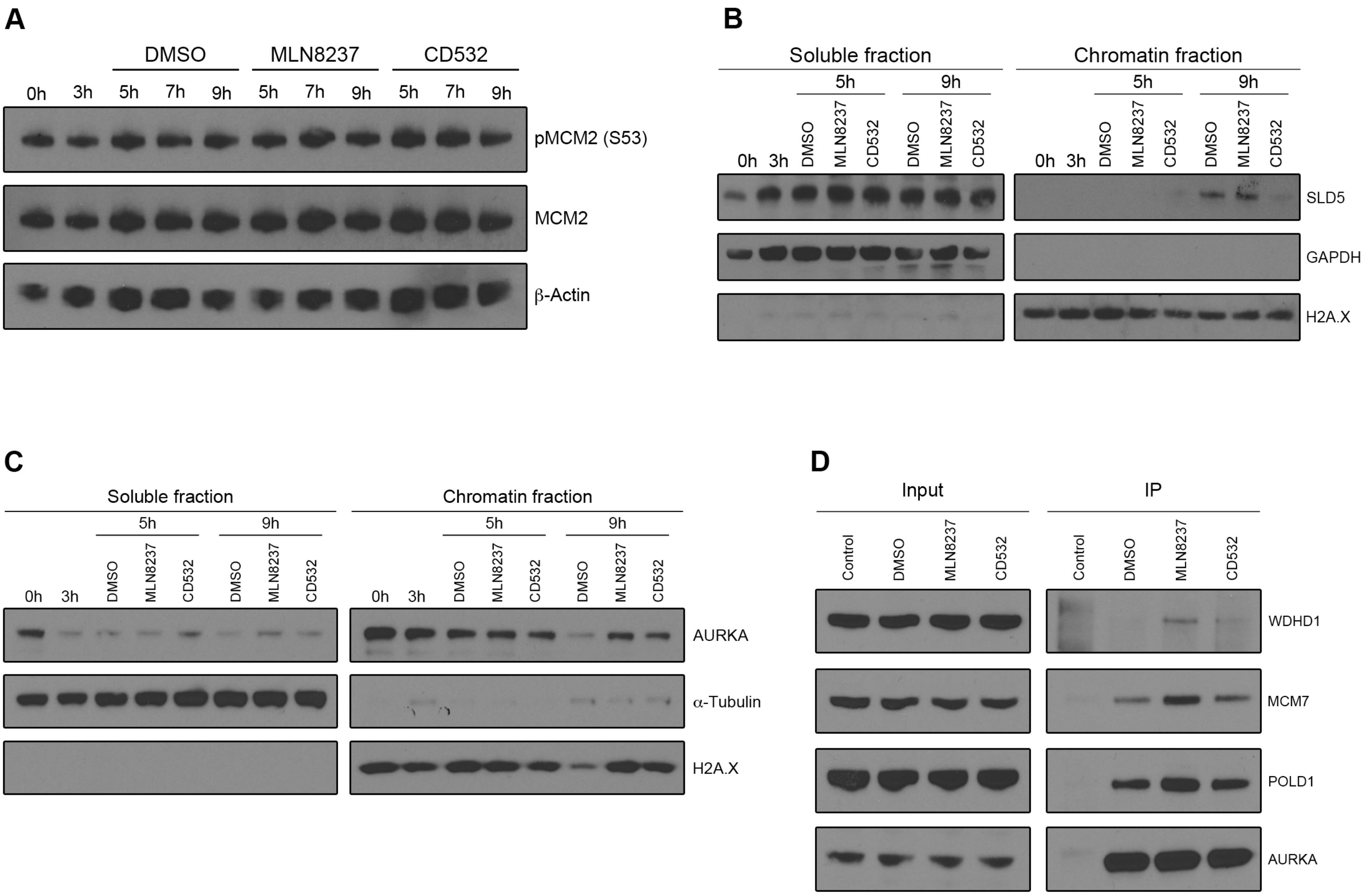

Supplementary Figure S5

A

	GI₅₀	*Fold difference*
**MLN8237**	49.7 nM	
**MLN8237 + PHA-767491 GI$_{25}$**	33.7 nM	1.4
**VX-689**	370.4 nM	
**VX-689+ PHA-767491 GI$_{25}$**	266.9 nM	1.3
**PHA-767491**	3.9 μM	
**PHA-767491 + MLN8237 GI$_{25}$**	2.9 μM	1.3
**PHA-767491**	3.9 μM	
**PHA-767491 + VX-689 GI$_{25}$**	3.7 μM	1.1

B

	GI$_{50}$	*Fold difference*
**CD532**	226.9 nM	
**CD532 + PHA-767491 GI$_{25}$**	46.8 nM	4.8
**PHA-767491**	3.9 μM	
**PHA-767491 + CD532 GI$_{25}$**	0.2 μM	19.5

C

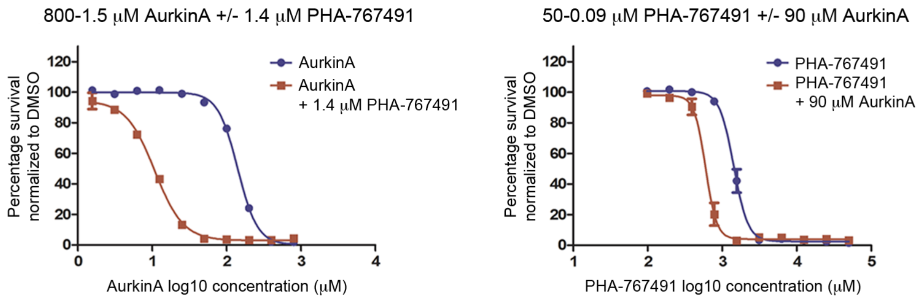

	GI$_{50}$	*Fold difference*
**AurkinA**	140.9 μM	
**AurkinA + PHA-767491 GI$_{25}$**	10.8 μM	13.0
**PHA-767491**	1.4 μM	
**PHA-767491 + AurkinA GI$_{25}$**	0.6 μM	2.3

D

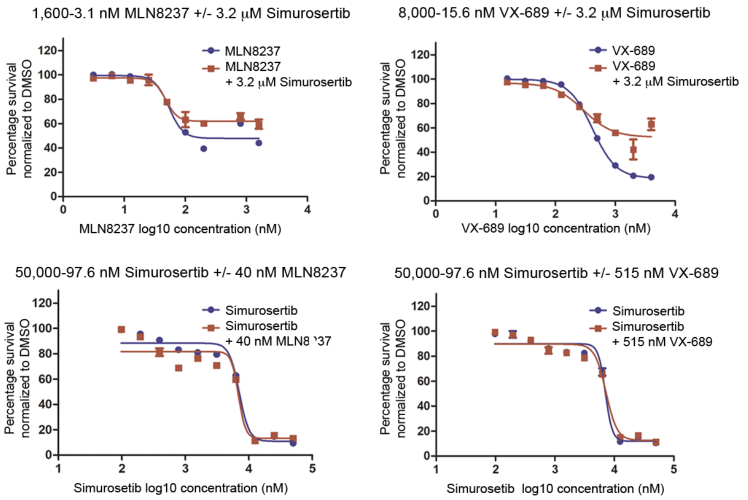

E

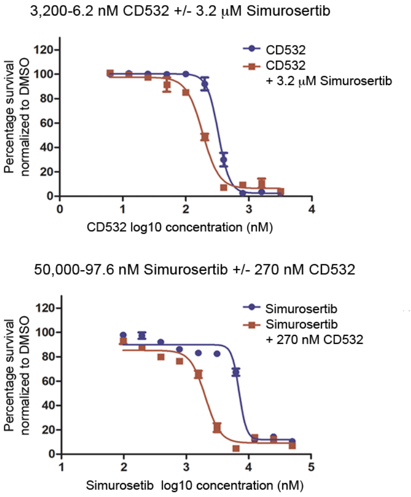

	GI$_{50}$	*Fold difference*
**MLN8237**	53.6 nM	
**MLN8237 + Simurosertib GI$_{25}$**	47.6 nM	1.1
**VX-689**	416.4 nM	
**VX-689+ Simurosertib GI$_{25}$**	293.9 nM	1.4
**Simurosertib**	7.0 μM	
**Simurosertib + MLN8237 GI$_{25}$**	4.7 μM	1.4
**Simurosertib**	7.0 μM	
**Simurosertib + VX-689 GI$_{25}$**	7.2 μM	1

	GI$_{50}$	*Fold difference*
**CD532**	329.1 nM	
**CD532 + Simurosertib GI$_{25}$**	185.1 nM	1.8
**Simurosertib**	7.0 μM	
**Simurosertib + CD532 GI$_{25}$**	2.0 μM	3.5
